# Fall-risk-increasing drugs and falls requiring health care among older people with intellectual disability in comparison with the general population: A register study

**DOI:** 10.1371/journal.pone.0199218

**Published:** 2018-06-19

**Authors:** Anna Axmon, Magnus Sandberg, Gerd Ahlström, Patrik Midlöv

**Affiliations:** 1 Occupational and Environmental Medicine, Department of Laboratory Medicine, Lund University, Lund, Sweden; 2 Department of Health Sciences, Lund University, Lund, Sweden; 3 Center for Primary Health Care Research, Department of Clinical Sciences in Malmö, Lund University, Lund, Sweden; University of Malaya, MALAYSIA

## Abstract

**Background:**

Falls are the most common cause of injury for older people in the general population as well as among those with intellectual disability. There are many risk factors for falls, including a range of drugs which are considered to be fall-risk-increasing (FRIDs). The aim of the present study was to describe prescription patterns of FRIDs in itself as well as in relation to falls requiring health care among older people with intellectual disability and their age-peers in the general population. Moreover, to investigate possible differences between the two groups.

**Methods:**

A cohort of people with intellectual disability and a referent cohort, one-to-one-matched by sex and year of birth, were established. Each cohort comprised 7936 people aged 55+ years at the end of 2012. Register data were collected for 2006–2012 on prescription of antidepressants, anxiolytics, hypnotics and sedatives, opioids, and antipsychotics, as well as for fall-related health care contacts. Analyses were performed on yearly data, using repeated measures models.

**Results:**

People with intellectual disability were more likely to be prescribed at least one FRID (Relative Risk [RR] 2.31). The increase was highest for antipsychotics (RR 25.0), followed by anxiolytics (RR 4.18), antidepressants (RR 2.72), and hypnotics and sedatives (RR 1.42). For opioids, however, a lower prevalence (RR 0.74) was found. In both cohorts, those with prescription of at least one FRID were more likely to have a fall-related injury that required health care. The increased risk was higher in the referent cohort (RR 3.98) than among people with intellectual disability (RR 2.27), although people with intellectual disability and prescription still had a higher risk of falls than those with prescription in the referent cohort (RR 1.27). A similar pattern was found for all drug groups, except for opioids, where prescription carried the same risk of having a fall-related injury that required health care in both cohorts.

**Conclusions:**

With or without prescription of FRIDs, older people with ID have a higher risk of falls requiring health care than their age-peers in the general population. It is important to be aware of this when prescribing drugs that further increase the risk of falls.

## Introduction

Falls are the most common cause of injury in the general older population [1–3] as well as among people with intellectual disability (ID) [3–6]. In comparison with the general population, people with ID are at increased risk of falls, fall-related fractures, and other fall-related injuries [4, 6–10], and the association between falls and injuries is stronger [3]. There are several possible explanations for this discrepancy, many of which relate to personal and health factors among people with ID, such as epilepsy or other seizure disorders [4, 5, 11], behavioral problems [5], and urinary incontinence [4].

In the general population, a range of drugs have been identified as fall-risk-increasing drugs (FRIDs), e.g. antidepressants, anxiolytics, hypnotics and sedatives, and antipsychotics [12–17]. All of these drugs are commonly prescribed to people with ID [18, 19]. Thus, a higher use of FRIDs could partly explain the increased risk of falls and fall-related injuries among people with ID. To date, no results regarding differences in prescription or use of FRIDs as a potential pathway to differences in falls are available in the scientific literature, although a conference presentation by Foran et al [20] indicates a possible association between drug use and risk of falling among people with ID.

Apart from prescription of FRIDs, a variety of medical conditions which are common among people with ID could explain the discrepancy in falls and fall-related injuries. These include epilepsy [21, 22], psychosis [23, 24], depression [25, 26], heart disease and cardiovascular disorders [10, 25], pain [10, 27], dementia and other cognitive impairments [25, 28], Parkinson disease [25, 29], visual impairments [10, 25], and diabetes [25, 30]. Differences in prevalence of such medical conditions should be taken into consideration when assessing the effect of FRIDs on risk of falls among people with ID in comparison with the general population.

The aim of the present study was to describe prescription patterns of FRIDs in itself as well as in relation to falls requiring health care among older people with ID and their age-peers in the general population. We also investigated possible differences between the two groups.

## Materials and methods

### Data sources

This is a register-based study and uses four Swedish national registers. The registers and their use in the study have been described in detail previously [31]. Briefly, the **LSS register**, which contains information on support and services provided to people with ID and/or autism spectrum disorder, was used to identify a cohort of people with ID (the ID cohort) aged 55+ years and alive at the end of 2012 (n = 7936). The **Total Population Register**, in which life events of the Swedish population are collected, was used to establish a reference cohort from the general population (the gPop cohort), one-to-one matched on birth year and sex. Data regarding prescriptions of FRIDs were collected from the Swedish **Prescribed Drugs Register** for the period 2006–2012. This register contains information on all dispensed prescriptions in Sweden based on the Anatomical Therapeutic Chemical (ATC) classification system [32]. The ATC system consists of five levels, where the fifth level identifies the chemical substance. The ATC classification system also includes Defined Daily Dose (DDD) for many drugs, where the DDD is the average adult dose used for the main indication of the medicine. Finally, the **National Patient Register**, which contains information on inpatient care episodes and outpatient specialist visits, with diagnoses recorded according to ICD-10 (International Statistical Classification of Diseases and Related Health Problems 10th Revision), was used to identify fall-related health care contacts during 2006–2012. These were used as a proxy for falls.

### Fall-risk-increasing drugs and health care contacts due to falls

In 2010, the Swedish National Board of Health and Welfare published a report listing drugs that may need extra attention among older people [1]. One group of such drugs were those that may increase the risk of falls. This group included drugs that may cause orthostatic hypotension (ATC-codes C01D, C02, C03, C07, C08, C09, G04CA, N04B, N05A excluding N05AN, and N06A), opioids (N02A), antipsychotics (N05A excluding N05AN), anxiolytics (N05B), hypnotics and sedatives (N05C), and antidepressants (N06A). In the present study, we included those drugs acting on the nervous system (ATC-code N), i.e. antidepressants (N06A), antipsychotics (N05A), anxiolytics (N05B), hypnotics and sedatives (N05C), and opioids (N02A). We compared the ID cohort with the gPop cohort with respect to a) having at least one prescription of each drug during each year, b) number of years with prescription, and c) individual average DDD per drug during years with prescription. These analyses were performed for the whole cohorts, as well as stratified by sex.

Through the National Patient Register, we identified all health care contacts that were registered as due to falls (diagnostic codes W00-W19 in ICD-10). We will henceforth refer to these as “fall”. We compared those with at least one prescription of each FRID during each year in the ID cohort to those with at least one prescription of that FRID in the gPop cohort with respect to falls during that year.

### Potential confounders

In order to evaluate whether potential differences in falls between the ID and gPop cohorts could–at least partly–be explained by discrepancies in fall-risk-increasing conditions, we collected information on such conditions from the National Patient Register. Conditions included were epilepsy (G40-G41 in ICD-10, n = 1300 in the ID cohort and n = 81 in the gPop cohort), psychosis (F20-F29; n = 426 and n = 43), depression (F32-F33; n = 413 and n = 312), dysrhythmia (I44-I49; n = 362 and n = 555) and heart failure (I50; n = 359 and n = 236), pain (M25.5, M54.5, M54.6, M79.6, R07, R10, R30, and R52; n = 1730 and n = 2275), dementia and other cognitive impairments (F00-F05; n = 94 and n = 53), Parkinson disease (G20-G22; n = 71 and n = 28), visual impairments (H0-H4; n = 1525 and n = 1110), and diabetes (E10-E14; n = 801 and n = 652).

### Statistics

Analyses of dichotomous outcomes (e.g. having at least one prescription) were performed using generalized linear models (GLM) with a Poisson distribution and log link, thus estimating relative risks (RRs) with 95% confidence intervals (CIs). The two cohorts were compared with respect to prescription of different groups of FRIDs. Moreover, among those with prescription, cohort comparisons were made with respect to risk of falls. Both these analyses were performed using yearly observations, using calendar year to indicate repeated measures.

To investigate whether potential differences in fall risk was a reflection of overall differences in fall-risk between people with ID and the general population, we evaluated the interaction effect between having prescription of FRIDs and cohort affiliation on having a fall. This was done by adding an interaction term to the GLM.

In the analyses of cohort differences in risk of prescription on falls, potential confounding of fall-risk-increasing disorders was assessed by entering each diagnosis, one by one, into the model. Confounding was considered present if the RR for falls changed at least 10% when the diagnosis was included.

P-p-plots revealed that although the original values of individual average DDD were skewed, ln-transformed values were normally distributed. Thus, analyses were performed using Analysis of Variance (ANOVA) on ln-transformed values. As data regarding number of years with prescriptions were skewed both in their original form and after ln-transformation, comparisons were made using the Mann-Whitney U-test.

A two-tailed p-value of 0.05 was considered statistically significant. All statistical analyses were performed in IBM SPSS Statistics version 23.

### Ethics

Approval was obtained from the Regional Ethical Review Board in Lund (reg no 2013/15). The National Board of Health and Welfare performed a separate secrecy review in 2014 before providing access to the data. All analyses were performed using anonymized datasets.

Data in the present study are based on anonymized information provided by two official government agencies: The National Board of Health and Welfare and Statistics Sweden. These authorities provide anonymized information for research purposes to individual researchers once the study has been vetted and approved by the Regional Ethical Review Board according to Swedish ethical review regulations. Due to the requirement of anonymized data, each individual could not be asked for consent to participate; active refusal of participation was instead applied. This was done by publishing information about the planned study in the Swedish national newspaper”Dagens Nyheter” and in UNIK, the magazine of The Swedish National Association for People with Intellectual Disability (FUB). The target audience for the UNIK magazine is mainly members (people with ID) and their families. Two versions of the advertisement were written, whereof one was an easy-to-read text. The advertisement presented the study and contained information on how to contact the research manager (GA) by phone, email or mail in order to opt out of the study. The research manager was then responsible for contacting the two national government agencies so that those who opted out were excluded before the authorities provided any data to the research manager.

## Results

Of the 7936 individuals included in each cohort, 3609 (45%) were women and 4327 (55%) were men. The age at the start of the study period, i.e. in 2006, ranged between 49 and 90 years, with a median age of 57 years. In the ID cohort, 5794 people (73%) had at least one FRID prescribed during the study period, and 2173 (27%) had at least one fall. The corresponding numbers in the gPop cohort were 4193 (53%) for prescription of FRIDs and 1139 (14%) for falls.

### Drug prescriptions

Using yearly data, people in the ID cohort were more likely than those in the gPop cohort to be prescribed at least one FRID (**Table 1**). The higher prevalence was found for all investigated FRIDs except opioids, for which the opposite prescription pattern was found. The largest increase was found for antipsychotics, followed by anxiolytics, antidepressants, and hypnotics and sedatives. When stratified by sex, a similar pattern emerged as when analyzing the whole cohorts. However, the higher prevalence associated with ID was consistently higher among men than among women.

**Table 1:** Cohort comparison of prescription of fall risk-increasing drugs (FRIDs).

	All (n = 7936)^1^	Women (n = 3609)^1^	Men (n = 4327)^1^
At least one FRID	2.31 (2.23–2.38)	1.93 (1.85–2.02)	2.78 (2.64–2.92)
Antidepressants	2.72 (2.55–2.91)	2.23 (2.05–2.43)	3.53 (3.18–3.91)
Anxiolytics	4.18 (3.90–4.49)	3.33 (3.03–3.66)	5.42 (4.86–6.05)
Hypnotics and sedatives	1.42 (1.32–1.51)	1.13 (1.03–1.24)	1.84 (1.66–2.03)
Opioids	0.74 (0.69–0.79)	0.76 (0.69–0.83)	0.71 (0.64–0.78)
Antipsychotics (previously presented in [33])	25.0 (21.3–29.4)	NC^2^	27.8 (22.2–34.8)

^1^ Numbers are given for each cohort.

^2^ Not calculated as the number of women in the gPop cohort with prescription was too low.

Risk ratios with 95% confidence intervals for prescription of fall risk-increasing drugs (FRIDs) for people with intellectual disability vs a one-to-one age and sex matched sample from the general population.

Among those with at least one prescription in the ID cohort, prescription during the entire study period was common, with 60% having at least one prescription of FRIDs during each year compared to 19% in the gPop cohort. This resulted in a higher median number of years with prescription in the ID cohort for all FRIDs with the exception of opioids (**Table 2**). The results were consistent when stratified by sex.

**Table 2:** Prescribed amount and number of years of prescription of fall-risk-increasing drugs during the study period (2006–2012).

	All			Women			Men		
	DDD		Years	DDD		Years	DDD		Years
	n	Geometric mean (range)	Median (range)	n	Geometric mean (range)	Median (range)	n	Geometric mean (range)	Median (range)
**At least one FRID**									
gPop	4157	55 (1-3403)	3 (1-7)	2136	61 (1-2788)	3 (1-7)	2021	50 (1-3403)	2 (1-7)
ID	5787	164 (1-3121)	7 (1-7)	2707	160 (1-2642)	7 (1-7)	3080	168 (1-3121)	7 (1-7)
ID vs gPop (p)^1^		**<0.001**	**<0.001**		**<0.001**	**<0.001**		**<0.001**	**<0.001**
**Antidepressants**									
gPop	1523	160 (3-3052)	3 (1-7)	888	158 (3-1609)	3 (1-7)	635	163 (3-3052)	2 (1-7)
ID	2766	302 (0-3121)	7 (1-7)	1403	299 (2-1408)	7 (1-7)	1363	304 (0-3121)	7 (1-7)
ID vs gPop (p)^1^		**<0.001**	**<0.001**		**<0.001**	**<0.001**		**<0.001**	**<0.001**
**Anxiolytics**									
gPop	1331	25 (1-3900)	2 (1-7)	767	23 (1-1549)	2 (1-7)	564	28 (1-3900)	1 (1-7)
ID	3322	53 (1-2223)	3 (1-7)	1583	47 (1-2063)	3 (1-7)	1739	58 (1-2223)	3 (1-7)
ID vs gPop (p)^1^		**<0.001**	**<0.001**		**<0.001**	**<0.001**		**<0.001**	**<0.001**
**Hypnotics and sedatives**									
gPop	1823	86 (3-2524)	3 (1-7)	1030	86 (3-2500)	3 (1-7)	793	87 (5-2524)	2 (1-7)
ID	2201	147 (3-2642)	4 (1-7)	1036	145 (3-2642)	4 (1-7)	1165	149 (3-1931)	4 (1-7)
ID vs gPop (p)^1^		**<0.001**	**<0.001**		**<0.001**	**<0.001**		**<0.001**	**<0.001**
**Opioids**									
gPop	2761	20 (1-2788)	1 (1-7)	1356	20 (1-2788)	1 (1-7)	1405	19 (1-1057)	1 (1-7)
ID	2051	21 (1-2831)	1 (1-7)	1054	22 (1-1474)	1 (1-7)	997	20 (1-2831)	1 (1-7)
ID vs gPop (p)^1^		**0.038**	0.96		0.081	0.90		0.27	0.63
**Antipsychotics** (previously presented in [33])									
gPop	235	38 (1-1154)	2 (1-7)	116	31 (1-706)	2 (1-7)	119	47 (2-1154)	2 (1-7)
ID	3116	122 (1-2336)	7 (1-7)	1337	109 (1-1932)	7 (1-7)	1779	133 (1-2336)	7 (1-7)
ID vs gPop (p)^1^		**<0.001**	**<0.001**		**<0.001**	**<0.001**		**<0.001**	**<0.001**

^1^ DDDs are compared using Analysis of Variance (ANOVA) on ln-transformed values, and number of years are compared using the Mann-Whitney U-test.

Prescribed amount measured as Defined Daily Dose (DDD) and number of years with prescription of fall-risk-increasing drugs (FRIDs) during the study period among 7936 people with intellectual disability (ID) and a sample from the general population (gPop) one-to-one matched on sex and age.

Among those with at least one prescription of each respective FRID, people in the ID cohort were prescribed higher annual doses (measured as DDDs) for each year with a prescription than people in the gPop cohort (**Table 2**). Again, the only exception was opioids.

### Prescriptions vs falls

Using yearly data, those with prescriptions of any FRID in both the ID and gPop cohort were more likely to have a fall (**Table 3**). With the exception of antipsychotics, an increased risk of falls was associated with ID cohort affiliation for all FRIDs. A possible interaction between cohort affiliation and having at least one prescription of FRIDs during the study period was found for all FRIDs except opioids. The interaction was consistently such that prescription indicated higher risk of falls in the gPop cohort than in the ID cohort.

**Table 3:** Relative risks with 95% confidence intervals for falls.

	ID vs gPop		Prescription vs no prescription		Interaction
	Prescription	No prescription	ID	gPop	p
At least one FRID	1.27 (1.16–1.39)	2.22 (1.98–2.49)	2.27 (2.08–2.48)	3.98 (3.56–4.44)	<0.001
Antidepressants	1.57 (1.33–1.86)	2.20 (2.03–2.39)	1.32 (1.21–1.44)	1.85 (1.58–2.17)	<0.001
Anxiolytics	1.41 (1.17–1.69)	2.14 (1.97–2.32)	1.40 (1.28–1.52)	2.11 (1.77–2.52)	<0.001
Hypnotics and sedatives	1.66 (1.40–1.97)	2.29 (2.11–2.48)	1.36 (1.24–1.50)	1.88 (1.60–2.20)	0.001
Opioids	2.43 (2.19-2.70)	2.50 (2.24-2.68)	6.36 (5.89-6.87)	6.41 (5.76-7.13)	0.91
Antipsychotics	1.00 (0.70–1.42)	2.17 (2.00–2.35)	1.11 (1.02–1.21)	2.41 (1.70–3.42)	<0.001

Risk ratios with 95% confidence intervals for having at least one fall during the year for people with intellectual disability (ID) compared with a one-to-one age and sex matched sample from the general population (gPop) stratified by prescription of fall-risk-increasing drugs (FRIDs), and for having at least one prescription of FRIDs during the year stratified by cohort. p-values refer to interaction between cohort (ID or gPop) and prescription of each respective drug group.

Potential confounding of fall-risk-increasing disorders was assessed among those with prescription of FRIDs. In these cohort comparisons, adjusting for diagnosis of epilepsy lowered the crude RRs for FRIDs vs falls. When adjusting for diagnosis of epilepsy, the RR for falls among ID vs gPop was 1.07 (95% CI 0.97–1.18) for at least one FRID, 1.36 (1.16–1.61) for antidepressants, 1.13 (0.93–1.36) for anxiolytics, 1.42 (1.19–1.69) for hypnotics and sedatives, 2.18 (1.95–2.43) for opioids, and 0.89 (0.62–1.27) for antipsychotics. Adjusting for the other fall-risk-increasing disorders did not change the effect estimates more than marginally (all <10%; data not shown).

## Discussion

Older people with ID are more likely than their age peers in the general population to be prescribed FRIDs. They are also prescribed FRIDs at higher doses and for longer durations. Furthermore, people with ID that have been prescribed FRIDs are more likely than those in the general population to fall.

People with ID were, with the exception of opioids, more likely than their age-peers in the general population to be prescribed all FRIDs that were analyzed in our study. They were also more likely to be prescribed higher doses and for a longer duration. In comparison with the general population, people with ID have higher occurrence of both somatic [10] and psychiatric [24, 34] diagnoses. Thus, that higher prescription rates should be found in this group is not surprising. However, the size of the increased frequency of prescriptions found in the present study does not correspond to the increased risk of psychiatric diagnoses found in the same population [24]. Hence, it cannot be ruled out that there is an over-prescription of FRIDs among older people with ID. Indeed, several studies have suggested that off-label prescriptions are common to treat challenging behaviors among people with ID [19, 35, 36]. This is worrisome as FRIDs have other adverse effects, not just than an increased fall-risk, especially among older people [1, 37]. In Sweden, it is mandatory for health care providers to offer medication reviews to people aged 75+ years and with prescriptions of at least five drugs [38]. People with ID have been suggested to age at an earlier chronological stage [39]. Considering this, and that people with ID as a rule are prescribed more drugs and have higher disease burden than the general population, it would be prudent to start medication reviews even earlier in this population to ensure that all prescribed medications correspond to a correct and current indication. This is important for all medications, but for FRIDs in particular.

People with ID had an increased risk of falls compared with the general population. Somewhat surprisingly, this increase could not be explained by fall-risk increasing disorders prevalent among people with ID, other than epilepsy. Furthermore, even after adjusting for diagnosis of epilepsy, the fall-risk pattern was similar to the crude one, although slightly less pronounced. This highlights the complexity of falls and fall-risk factors among older people with ID. Nevertheless, among people with ID, as in the general population, falls may be prevented by exercises and physical activity [40, 41]. Thus, in addition to medication reviews, physiotherapy interventions are relevant among older people with ID in order to reduce falls.

In general, FRIDs implied a higher fall-risk among people in the general population than among people with ID. This is most likely not an indication of these drugs being less inappropriate among people with ID, but rather that the number of fall-risk factors are much higher in this population than among people without ID.

A major strength of the present study is the use of the Swedish Drug Prescription Register to obtain drug data. Starting July 2005, the register contains data concerning all purchases of prescribed drugs in Sweden [42]. All the FRIDs investigated in the present study are only sold via prescription in Sweden. Thus, no misclassification has been introduced by over-the-counter purchases. However, as only dispensed drugs are recorded, the information collected through the register is likely to be an underestimation of the number of prescriptions. Moreover, as purchase of a drug does not necessarily equal use (secondary non-adherence), register data is likely to be an overestimation of drug use. This needs to be considered in the interpretation of data.

In most populations, including the general Swedish population, the male-to-female ratio is generally in favor of women (i.e. below 1). However, in the present study, the opposite was found in the ID cohort (and consequently in the gPop cohort as well), i.e. there were fewer women than men. This raises the question of a possible skewness in the sex distribution in the present study. However, although declining with age, prevalence of ID is higher among men than women [43], resulting in a higher male-to-female ratio among people with ID than in the general population [44]. Thus, it should not be a cause of concern that the sex distribution in the ID cohort is skewed compared to the general population.

We used health care contacts in inpatient and outpatient specialist care, and registered as fall-related, as proxy for falls. This is, of course, an underestimation of all falls, as many falls may not lead to any health care contact or to a visit in primary care. This is also something that needs to be taken into consideration when interpreting the results from the present study.

As only people alive at the end of 2012 were included in the study, falls severe enough to cause death are not included in the analyses. Thus, the number of falls found in the present study may be an underestimation of the true numbers. Moreover, if falls are more likely to cause death among people with ID than in the general population, the cohort comparisons will be biased. To the best of our knowledge, no studies have been performed comparing people with ID to the general population regarding death after falling. However, external underlying causes of death overall (i.e. including also poisoning, accidents, etc.) seem to be more common among people with ID [45, 46].

When analyzing the association between prescription of FRIDs and occurrence of falls, we did not take into account the timing of the prescription in relation to the fall. A contributing reason for this approach was that we had no data on when the FRID was used, only when it was purchased. Moreover, that we had information only on a relatively short time window (seven years), and we could not discriminate those who had not been prescribed FRIDs prior to this period from those who had. Thus, we can make no statements about a possible causality of prescription of FRIDs and risk of falls, but only draw conclusions about associations between these two factors.

In the general population sample, approximately one fourth were prescribed at least one FRID each year. This is in agreement with prescription rates in the whole of Sweden [47], suggesting that the gPop cohort constitutes a representative sample from the general population.

## Conclusions

Older people with ID are more likely to be prescribed FRIDs and have a higher risk of falls requiring health care than their age-peers in the general population, even when taking into account differences in prevalence of fall-risk-increasing disorders. Moreover, they tend to have prescriptions for longer periods of time. Even without the use of FRIDs, older people with ID have a high risk of falls. It is important to be aware of this when prescribing drugs that increase the risk of falls further.
